# Stress responses in high-fidelity simulation and standard simulation training among medical students

**DOI:** 10.1186/s12909-023-04101-x

**Published:** 2023-02-17

**Authors:** Pamela Barbadoro, Agnese Brunzini, Jacopo Dolcini, Luca Formenti, Aurora Luciani, Daniele Messi, Alessandra Papetti, Elisa Ponzio, Michele Germani, Starlab Working Collaborative Group, Daniele Arsego, Elena Bianchi, Rossella De Angelis, Luca Del Bene, Rosaria Landi, Ferruccio Mandorli, Maria Rosaria Marcone, Rebecca Micheletti, Guido Paolucci, Mauro Pesaresi, Andrea Santarelli, Erica Adrario

**Affiliations:** 1 Department of Biomedical Sciences and Public Health, Section of Hygiene, Preventive Medicine and Public Health, Polytechnic University of the Marche Region, Ancona, Italy; 2 Department of Industrial Engineering and Mathematical Sciences, Polytechnic University of the Marche Region, 60131 Ancona, Italy; 3 Department of Biomedical Sciences and Public Health, Polytechnic University of the Marche Region, Ancona, Italy; 4 Anesthesia and Intensive Care Unit, Azienda Ospedaliero-Universitaria “Ospedali Riuniti”, Ancona, Italy

**Keywords:** Medical training, Stress, High-fidelity, Cortisol, Anxiety, Simulation

## Abstract

**Background:**

Simulation has been recognized as a shift in healthcare education that can improve skills and patient safety and outcomes. High-fidelity simulation of critical medical situations can be a source of stress among participants that can interfere with students' abilities leading to unexpected emotional responses. The aim of this study is to determine if two simulation methods, high-fidelity (HF) and procedural simulation (PS), may be associated with stress responses at a self-perceived and biological level (salivary cortisol variations), and to compare stress levels of the two different simulation method. We also wanted to find independent variables associated with cortisol total hormonal output.

**Methods:**

A quasi-experimental before-after study was used including the administration of questionnaires, and biomarkers evaluation by salivary cortisol samples before and after simulation. A total of 148 students were eligible and agreed to participate in the study. We used paired T-test for mean comparison regarding State-trait anxiety for both HF and PT simulations. For NASA-TLX we performed a T-test mean comparison between groups. We used paired T-test mean comparison for cortisol analysis. Multivariable linear regression has been used to assess variables associated with AUC_g_ values and perceived stress.

**Results:**

values of STAI-Y scores were relatively higher at the end of the HF and PS sessions. NASA-TLX was significantly higher at baseline for the HF simulations, with respect to the PS simulation. Cortisol fold increase was significantly different in the two groups. Linear regression showed that cortisol AUCg was related to the STAI-Y score in both groups.

**Conclusion:**

Participating students developed a stress response both after in the HF and PS training, testified by psychological and biological outputs. According to our results, stress levels were increased for simply being in a simulation scenario than to the intrinsic complexity of the task required. More studies are needed to confirm this trend and to clarify the role of simulated stress response in a long-term learning scenario.

## Background

Simulation can be defined as a technique of imitating the behaviour of a situation or process by means of a suitably analogous situation and is a learning activity that can easily imitate the reality of a patient and the traditional clinical setting. Moreover, simulation can be considered an educational strategy that paired with technology helps develop skills, competencies, and clinical judgement in a safe environment [1, 2].

According to the Society for Simulation in Healthcare (SSH), simulation has been recognized as a shift in healthcare education that can improve skills and patient safety and outcomes [3].

In this context, technological innovations, such as high-fidelity, augmented, and virtual reality simulation models and the use of simulated logical assistance paths, have led to the constant improvement of skills acquisition and forecast of possible consequences of healthcare and their management [4–6].

Moreover, during the design of the training activity, it should not be underestimated that participation in an engaging simulation, such as the high-fidelity one, can have a considerable psychological impact on the participants, causing high cognitive stress, especially in the most anxious subjects [7, 8].

A high-fidelity realistic simulation may expose participants to significant stress. Stress can be beneficial, increasing the cognitive emergency response and allowing acute adaptation, but can also lead to unexpected reactions (screaming, aggressiveness, or complete inhibition) and sometimes it may alter the performance. Some studies found impaired performances in health care workers under acutely stressful situations [7], but others found improved performances [8].

High-fidelity simulation of critical medical situations can be a source of stress and anxiety among participants, as suggested by several studies. The intensity of stress interferes with students' technical and non-technical abilities and can sometimes lead to unexpected emotional responses [9, 10].

The aim of this study is to determine stress responses at a self-perceived and biological level (salivary cortisol variations), of two simulation methods, high-fidelity (HF) and procedural simulation (PS), and to compare elicited stress levels of the two different simulation methods. We also wanted to find independent variables associated with cortisol total hormonal output and perceived stress.

## Methods

### Study design

A quasi-experimental before-after study was used to study the stress response during two different training sessions (HF and PS); the study included the administration of questionnaires before and after simulation, and biomarkers evaluation by salivary samples, followed by a debriefing session. The sample size was chosen to obtain a significance level of 5% and a study power of 80%.

After completing the pre-test questionnaire, participants proceeded directly to the simulation-based team training. The sessions included: a) a high-fidelity emergency training situation (HF Simulation) and b) procedural simulation (PS, i.e. the performance of a complex neurological technique such as spinal tap employing). The two simulations have been performed in two separate days.

Before participating, students were adequately informed about the objective of the study and the possibility to drop at any time. Those who decided to participate signed consent for participation and processing of personal/biological data. Each student has been assigned to a recognition code, which allowed the anonymization of the data throughout the study.

The study was approved by the LOCAL Ethics Committee of the Polytechnic University of Marche (Prot. n. 0000197) and was carried out following the ethical standards required for biological rhythm research studies on human beings [11].

### Psychometric measures

A State-Trait Anxiety Inventory (STAI-Y) questionnaire, was administered before (T0) and after (T1) the HF and PS sessions to assess the current basal state of anxiety and its variations after the training sessions, respectively. Moreover, the NASA-Task Load Index (NASA-TLX), was administered at the end of the simulations to assess the mental workload. NATA-TLX is a multidimensional assessment tool measuring self-perceived workload in a task, system, etc.; it was developed by the Human Performance Group at NASA's Ames Research Center.

### Sample collection and Salivary cortisol detection

Smokers, obese (BMI > 30) students, those affected by chronic diseases or oral pathologies, taking beta-blockers, diuretics, or glucocorticoids, or those reporting bereavement or major stressful events in the previous six months were excluded. Before (T0) and after (T1) the training sessions, five saliva samples were obtained for determining a profile of salivary cortisol basal secretion. Salivary samples were collected at 5-time points across the sessions: upon the arrival of the student, 10 min before starting the simulation, and then at 10, 20, and 30 min after the training. For each sample, 1 ml of saliva was collected in Salivette®(Sarsted Aktiengesellschaft & Co., Nümbrecht, Germany); sampling procedures were carried out as previously described [12, 13]. Participants refrained from eating, drinking, smoking, and brushing for at least 30 min before sample collection. Saliva samples were centrifuged at 1000 g for 2 min to produce a clear supernatant of low viscosity that was stored at − 20 ◦C. A commercial enzyme immunoassay kit to determine salivary cortisol (DRG Instruments GmbH, Germany) was used according to the manufacturer's instructions. All measurements were performed in duplicate. The intra-and inter-assay coefficients of variation were 4 per cent and 5 per cent. The assays were performed at the Unit of Hygiene, Preventive Medicine, and Public Health of the Polytechnic University of Marche. Cortisol concentration was expressed as nmol/l. All data are reported as mean ± standard error of means (SEM). We compared values of basal cortisol at arrival with several salivary samples collected after simulation used to calculate the 'Area under the curve with respect to the ground' (AUC_g_) as done by previous authors [14]. It can be assumed that AUC_g_ will result in a measure that is related to ‘total hormonal output’. Moreover, fold increase has been calculated as the ratio between cortisol level at 10 min after the start of training and the basal value.

### Statistical methods

We compared results coming from questionnaires and cortisol levels between the two simulation HF and PS that defined our groups.

We used paired T-test for mean comparison regarding State-trait anxiety for both HF and PT simulations. For NASA-TLX we performed a T-test mean comparison between groups. We used paired T-test mean comparison for cortisol analysis. Multivariable linear regression has been used to assess variables associated with AUC_g_ values and perceived stress.

## Results

### Participants’ characteristics

A total of 170 students were screened for eligibility. Of these, 148 were eligible and agreed to participate in the study (63 men and 85 women; Mean age = 25.8 ± 0.08 years; Mean BMI = 21.8 ± 0.28).

### Psychometric measures

#### State-Trait Anxiety Inventory scores (STAI-Y)

Mean values at State-Trait Anxiety Inventory scores (STAI-Y) focusing on the Y1 scale were relatively higher at the end of the HF simulation session (mean score 45.13, 95%CI 44.46–45.90) than before the training session (mean score 44.13, 95%CI 43.37–44.9). Similar results have been registered in the STAY-Y score variations in the ST group, with a pre-training mean of 43.63, (95%CI 42.77–44.50) versus a mean of 44.63 (95%CI 43.83–45.43) after the simulation. No significant difference in STAI-Y scores has been registered between the two procedures.

#### NASA task load index (NASA-TLX)

Considering the NASA task load index (NASA-TLX), it was significantly higher at baseline for the HF simulations (mean score = 65.8, 95%CI, 63.01–68.52), with respect to the ST simulation (ST, *N* = 142, mean score = 52.5, 95%CI 49.4–55.6).

#### Salivary cortisol

Of the 148 subjects, 77 (52.02%) agreed and correctly collected all salivary samples. Mean salivary cortisol levels were similar in both groups (Table 1).

**Table 1:** Distribution of mean salivary cortisol levels in the two study groups

MEASURES	HF simulation Mean	95% C.I	ST simulation Mean	95% C.I
Salivary cortisol (nmol/l)	7.85	6.97- 8.73	6.12	5.24—6.99
*AUC*_*G*_ after simulation	28.99	26.19- 31.79	24.93	22.11—27.74
Fold Increase	1.46	1.23–1.69	1.22	1.04–1.40

Cortisol fold increase was significantly different in the two groups (mean 1.46, 95%CI 1.23–1.69 in the HF simulation versus 1.22, 95%CI 1.04–1.40 in the PS-simulation, paired t-test *p* < 0.05).

Cortisol AUCg was related to the STAI-Y score in both groups (coeff. 0.57, 95%CI 0.42–0.72 in HF-simulation, versus coeff. 0.50, 95%CI 0.33–0.68 in PS simulation); moreover, NASA-TLX was not significantly related to total cortisol level measured during the session; the hour of the day was related to AUCg only in HF group (coeff. -5.85, 95%CI -10.41–1.29) while sex was not related to AUCg in both groups (Table 2). We then ivestigated if same variabes could be related to perceived stress measured with the difference between STAI-Y scores before and after simulations. None of the aforementioned variables reached statistical significance (Table 3).

**Table 2:** Linear regression of HF and PS simulation AUCg values versus STAI-Y, Sex, Session and NASA-TLX score

	**Coeff** **(HF)**	**P-value** **(HF)**	**95% Confidence Interval** **(HF)**	**Coeff** **(PS)**	***P*-value** **(PS)**	**95% Confidence Interval** **(PS)**
STAI-Y	0.57	0.00	0.42; 0.72	0.50	0.00	0.33; 0.68
Sex	-3.32	0.15	-7.84; 1.19	-0.76	0.77	-5.87; 4.35
Session	-5.85	0.01	-10.41; -1.29	-1.84	0.48	-7.08; 3.39
Nasa-TLX	0.09	0.15	-0.03; 0.21	-0.04	0.52	-0.17; 0.08

**Table 3:** Linear regression of HF and PS simulation STAI-Y scores versus Sex, Session and NASA-TLX score

	**Coeff** **(HF)**	***P*-value** **(HF)**	**95% Confidence Interval** **(HF)**	**Coeff** **(PS)**	***P*-value** **(PS)**	**95% Confidence Interval** **(PS)**
Sex	1.56	0.2	-0.88; 4.01	0.16	0.89	-2.29; 2.61
Session	1.02	0.41	-1.45; 3.49	-0.97	0.4	-3.43; 1.49
Nasa-TLX	-0.04	0.25	-0.11:0.03	0.05	0.13	-0.01; 0.11

## Discussion

Task performance imposes high workload and personal concerns, which is why it may produce stress responses indicated by psychological, physiological, and humoral indices [15]. The objective of this study was to determine whether two simulations, HF and PS, were associated with stress responses as well as to find whether psychological aspects like anxiety and workload perceived levels may be associated with cortisol increase after completing the tasks. Our results showed that from a subjective point of view students perceived the HF simulation as more work loading compared to the PS simulation. Interestingly, students felt more anxious at the end of the simulation both in HF and PS showing that perceived anxiety could not be related to problem difficulty levels, quite in opposition to previous findings [16]. This may be due to the fact that trait anxiety is more influenced by participating in a general context of a training scenario where students are evaluated, rather than the kind, and complexity of training. Another interesting result of this study is that the total cortisol production, measured as AUCg levels, increased in both simulations compared to the basal levels of our subjects. So, in this case, the psychological stress component has been mirrored by a physiological hormone output. This result is quite in opposition to previous findings [17] but in accordance with what has been found by other studies where cortisol may reach its peak half an hour after a stressful event [18]. Again, increasing cortisol was not related to the kind of scenario since even if according to NASA-TLX scores the HF is more psychological demanding, we found a total hormone output increase in both simulations. Linear regression confirmed this aspect since AUCg resulted in positively associated with STAI-Y but not with NASA-TLX and negatively associated with afternoon session when levels of cortisol are physiologically lower but only in HF regression model this result reached statical significance. Another consideration that can be highlighted is that many studies have shown gender differences in stress responses, regulation, coping strategies [19–22] and in healthcare immersive simulations [23]. This is the reason why we performed our regression also considering gender in our models but for stress measured through cortisol metabolism with AUC_g_ values and perceived stress through STAI-Y scores, we didn’t find a significative association. Our study has several strengths since we have been able to evaluate students before and after two different types of simulation and the stress responses are in accordance both in terms of physiological stress outputs, measured through validated questionnaires, and both in terms of total cortisol production that it has been accurately evaluated collecting several saliva samples. This procedure takes into consideration the time variability of cortisol production, which is a fundamental component of the HPA axis response.

### Limitations

Our study has some limitations that we would like to highlight: first of all analysis of cortisol output has been performed through AUC_g_ values and not on actual cortisol levels. We opted for this method assuming that AUC_g_ is related to the ‘total hormonal output’, as done in previous studies [14]. Anyway, since AUC_g_ extrapolates on the evolution of the cortisol rate between peaks there is the possibility of an error margin that must be taken into consideration when looking at our results.Moreover, only a part of the total subjects who underwent simulations have been able to correctly collect a saliva sample and we did not analyze other biological stress elements like testosterone production, α-amylase, and secretory class-A immuno-globulins that have recently been investigated as possible stress sensors in similar studies [17]. Another important consideration that can be added is that the same students in our study performed the two simulations. This could have inevitably modified their primary state after performing the first simulation. To minimize this effect, to the best of our possibilities, we guaranteed an optimal debriefing performing the simulations on two different days with several days of rest between the two events. A possible improvement for the future could be to divide the students into two groups so that one group starts with the HF simulation and then continues with the PS simulation, and the other does the opposite and then compare the results.

## Conclusions

Results shown in this work highlight how our students developed a stress response from a subjective and objective point of view, testified by alteration of psychological and biological outputs. The simulations were stressful for our students and according to our results both the psychological and the biological aspects were modified due to simply being in a simulation scenario than to the intrinsic complexity of tasks required to be performed.More studies are needed to confirm this trend and to clarify the role of simulated stress response in a long-term learning scenario.

## Data Availability

The datasets used and/or analysed during the current study are available from the corresponding author on reasonable request.
